# Exploring the factors affecting the implementation of tobacco and substance use interventions within a secondary school setting: a systematic review

**DOI:** 10.1186/s13012-017-0659-8

**Published:** 2017-11-14

**Authors:** Gillian Waller, Tracy Finch, Emma L. Giles, Dorothy Newbury-Birch

**Affiliations:** 1 School of Health and Care, Health and Social Care Institute, Teesside University, Middlesbrough, TS1 3BA UK; 2 Institute of Health & Society, Newcastle University, Newcastle, NE2 4AX UK

**Keywords:** Systematic literature review, Implementation, Secondary school, Substance use, Tobacco, Normalisation Process Theory

## Abstract

**Background:**

The aim of this mixed-methods, systematic literature review was to develop an understanding of the factors affecting the implementation of tobacco and substance use intervention programmes in the secondary school setting using NPT as an analytical framework.

**Methods:**

A search strategy was developed that combined implementation, school and intervention search terms. Literature searches were conducted in MEDLINE, Embase, PsycHINFO, Scopus, ERIC, CINAHL, Web of Science and the Cochrane Library. PROSPERO was also searched for similar systematic reviews and a grey literature search of policy documents and relevant material was also conducted. Papers were eligible for inclusion if they were based in a secondary school and focused on the implementation of a tobacco or substance use programme. Both quantitative and qualitative methodologies were considered for inclusion. Normalisation Process Theory (NPT) was used as a conceptual framework to identify facilitators and barriers of implementation and to structure the synthesis.

**Results:**

Inclusion criteria were met by 15 papers. The included papers were both quantitative and qualitative and focused on a range of tobacco and substance use interventions, delivered by differing providers. Key facilitating factors for implementation were positive organisational climate, adequate training and teacher's and pupil’s motivation. Barriers to implementation included heavy workloads, budget cuts and lack of resources or support. Quality appraisal identified papers to be of moderate to weak quality, as papers generally lacked detail.

**Conclusion:**

NPT highlighted the need for studies to extend their focus to include reflexive monitoring around appraisal and the evaluation processes of implementing new tobacco or substance use programs. Future research should also focus on employing implementation theory as a tool to facilitate bridging the gap between school health research and practice.

**Electronic supplementary material:**

The online version of this article (10.1186/s13012-017-0659-8) contains supplementary material, which is available to authorized users.

## Background

Adolescence can be identified as a critical development phase and therefore a key stage within the life course. It is a time when adverse health-related behaviours, such as tobacco or substance use, are frequently established and ‘tracked’ into adulthood [1–4]. Adolescence—or the ‘secondary school years’—thus provides a key period for the delivery of interventions [2, 5–7] to deter uptake of unhealthy behaviours. The uptake of health risk behaviours are more likely to occur in the later stages of adolescence, between the ages of 15 and 19 years [8, 9]. This is largely due to the fact that a young person in late adolescence is more susceptible to social influences, such as peer pressure, experimentation and rebellion. These social influences are associated with an increased tendency to undertake in risk-taking behaviour, such as drug taking or risky alcohol consumption, and can play a substantial role in influencing long-term health outcomes [8, 10, 11].

As it remains compulsory for young people, in the UK, to engage in academic education until the age of 16 years; the secondary school setting acts as a platform in which to deliver preventative health education and complex interventions to tackle tobacco and substance use. Recent feasibility research exploring the delivery of brief alcohol interventions (ABIs) in secondary schools has proved effective, highlighting the potential as a setting to deliver such interventions [2, 7]. However, the effectiveness of school-based substance use interventions has often proved inconclusive. A specific example of this is a systematic review conducted by Foxcroft and Tsertsvadze, aiming to explore the extent of research around the effectiveness of school-based, alcohol primary prevention programmes [12]. The review identified that some studies showed no evidence of a primary intervention being effective in a school setting, whilst others presented statistically significant results, indicating a degree of effectiveness [12].

Breaking this down further, to assess whether complex substance use interventions have a place within secondary schools, although there remains to be a series of factors affecting the effectiveness of such an intervention, there does appear to be a gap between generating school-based, tobacco and substance use intervention research evidence and the implementation of this research in practice [13]. Very few of these papers offered a significant assessment of the factors affecting the implementation of their substance use interventions, or how varying the implementation process of such an intervention could have the potential to affect the effectiveness. Therefore, this systematic review was proposed as a way in which to collate the available evidence from studies, which present evidence around the factors affecting the implementation of a tobacco or substance use interventions, within a secondary school setting. It builds upon the current work in this area [14, 15], to provide an account of factors specific to secondary school level education and specifically the implementation of tobacco and substance use interventions.

The field of implementation science has been born as a result of recognising the importance of the gap between research and practice [13]. This gap has expedited the use of multitudinous theoretical constructs, aiming to enhance the implementation process, identify the barriers and facilitators and acting as valuable tools in evaluating implementation [16, 17]. Much of the advancing knowledge on barriers and facilitators to implementation has been construed within health care and the provision of primary care, and implementation theory has been frequently employed within this context [18, 19]. The use of theory has been less widely associated with school implementation research [20]. Therefore, this systematic review seeks to interpret and synthesise determinants of implementation in the school setting by using a specific implementation theoretical framework.

The Normalisation Process Theory (NPT) provides an explanation of the factors affecting whether an intervention can be incorporated into practice, with reference to the context in which the work of the new intervention occurs [21]. It focuses on understanding the implementation, embedding and integration of new technologies and organisational innovations by considering four theoretical constructs: Coherence, Cognitive Participation, Collective Action and Reflexive Monitoring [16, 21, 22]. Table 1 presents an overview of the four NPT constructs and its respective subconstructs.

**Table 1:** Normalisation Process Theory (NPT) breakdown of key constructs [16, 21, 22]

NPT construct	Definition	Sub constructs
Coherence	The sense-making work that people do individually and collectively when they are faced with the problem of operationalizing a set of practices.	• Differentiation • Communal specification • Individual specification • Internalisation
Cognitive Participation	The relational work that people do to build and sustain a community of practice around a new technology or a complex intervention.	• Initiation • Enrolment • Legitimation • Activation
Collective Action	The operational work that people do to enact a set of practices, whether these represent a new technology or a complex healthcare intervention.	• Interactional Workability • Relational Integration • Skill set Workability • Contextual Integration
Reflexive Monitoring	The appraisal work that people do to assess and understand the ways that a new set of practices affect them and the others around them.	• Systematisation • Communal appraisal • Individual appraisal • Reconfiguration

NPT is our chosen framework as it has demonstrated value in synthesising research findings to identify knowledge consistencies and gaps regarding implementation determinants [23, 24]. Although NPT was designed for implementation and integration problems in healthcare, the constructs are transferable and thus can be applied fluidly to consider the review’s focus of factors affecting implementation in the school setting [25]. As this field is currently small and studies of implementation are heterogeneous, NPT offers advantage as a theoretical framework for integrating both qualitative and quantitative findings to develop an assessment of the factors which can affect implementation in this context [25]. To our knowledge, NPT has not previously been used to synthesise findings in the context of secondary school implementation research.

### Aim

The aim of this systematic review was to identify and synthesise the factors affecting the implementation of tobacco and substance use interventions in the secondary school setting, by applying the Normalisation Process Theory.

## Methods

Using a registered protocol (PROSPERO: CRD42016039354), systematic review methods were undertaken to identify eligible literature. Developing specific inclusion and exclusion criteria allowed the selection of papers.

### Study inclusion and exclusion criteria

Both quantitative and qualitative studies were eligible for inclusion. Papers were not excluded by their methodology alone, and to minimise the risk of bias, papers were not excluded by their background, ethnicity or language. To be included, papers had to be based within a secondary school or the international equivalent, and focusing on students aged between 11 and 18 years. Papers based outside the secondary school (e.g. primary, universities and community locations) were excluded. Included papers were those that reported any factors affecting the implementation of a tobacco or substance use intervention. Studies conducted pre-1980 were excluded due to subsequent school system reforms, which would likely limit the relevance of findings.

### Literature searches

The electronic databases, MEDLINE, Embase, PsycHINFO, Scopus, ERIC, CINAHL, Web of Science and the Cochrane Library, were searched using specific key words to obtain eligible papers, as shown in Table 2. Search terms were modified to accommodate the differences in the databases, and Boolean search terms and MeSH terms were employed to ensure all relevant literature was searched for.

**Table 2:** Search Terms for each Database

Databases	Search terms—*combined with ‘AND’*			
	School	Implementation	Intervention/change	Health
Cochrane Library EMBASE Via OVID ERIC Via EBSCO host Medline Via EBSCO host SCOPUS Web of Science Via Thomson Reuters	school*	implement* **OR** adopt* **OR** integrate* **OR** normali*	improvement* **OR** innovation **OR** knowledge* **OR** organisational change* **OR** quality improvement **OR** readiness to change* **OR** behaviour change* **OR** intervention* **OR** school based intervention*	health*
CINAHL Via EBSCO host	school*	implement* **OR** adopt* **OR** integrate* **OR** normali*	CINAHL Search Terms: Health Behaviour exp. **OR** Behavioural Changes **OR** Behaviour Modification exp. **OR** Health Education Key Words: improvement* **OR** innovation **OR** knowledge* **OR** organisational change* **OR** quality improvement **OR** readiness to change* **OR** behaviour change* **OR** intervention* **OR** school based intervention*	health*
PSYCHINFO Via EBSCO host	school*	implement* **OR** adopt* **OR** integrate* **OR** normali*	MeSH Terms: Behaviour Change **OR** Health Education **OR** School Based Intervention Key Words: improvement* **OR** innovation* **OR** knowledge* **OR** organisational change* **OR** quality improvement **OR** readiness to change* **OR** behaviour change* **OR** intervention* **OR** school based intervention*	health*

OR and AND denote the Boolean operators used

* denotes truncation symbol

Grey literature, such as national government school curricula and local government websites, were searched for via common search engines using key words reflecting the formal research strategy. Potentially relevant material was obtained and assessed using the same inclusion and exclusion criteria. In addition, the PROSPERO database was searched to identify whether any similar reviews had been conducted.

### Study selection

Screening was undertaken by two of the review authors (GW and DNB). In the first round of screening, GW assessed papers against the criteria by reviewing the title and abstract and DNB was responsible for double sifting 20% of the results. If papers appeared relevant, the second stage involved full papers being obtained, assessed and retained if they continued to meet the inclusion criteria. One hundred percent of the papers at the second stage were double sifted by DNB. Any screening discrepancies between reviewers were resolved by further discussion and a third reviewer (TF) was consulted if necessary.

### Data extraction

A data extraction form was developed and piloted on the first five studies. The information extracted from each paper was based around the following: *Paper Reference and Location—*The author information and which country the study had been conducted in, *Intervention—*Whether the intervention was an alcohol, drug or substance use intervention or a combination of some or all, *Study Population—*How many young people or providers participated in the study, *Study Design—*The methods the study employed, *Implementation Measurement—*What was measured in relation to implementation, *Data Analysis*—What methods were used to analyse the collected data, and the *Key Results—*These were the key results stated in the paper that specifically identified as factors affecting implementation. The information extracted was used to formulate a summary table, which is displayed in Table 3, Additional file 1.

### Data synthesis

A narrative approach to synthesis was undertaken due to the expected heterogeneity of the included studies. The Normalisation Process Theory (NPT) [22, 25] was used as a novel way to structure the synthesis and to guide the assessment of established implementation factors reported in the included studies. NPT-based interpretations of the study findings were assessed by two authors (GW and TF) and discussed as necessary within the wider review team.

### Quality assessment

Included papers were assessed using quality assessment tools appropriate to the study design. However, due to the limited availability of relevant literature, quality was not used as an indicator of exclusion. The Effective Public Health Practice Project (EPHPP) appraisal tool was used to assess quantitative studies [26]. Each component of a paper was rated, with each rating being combined to obtain a global rating of Strong, (0 weak ratings), Moderate (1 weak) or Weak (2+ weak). The Critical Appraisal Skills Programme (CASP) tool was employed to appraise qualitative papers [27]. CASP assessed the included papers with three questions: Is the study valid? What are the results? and Are the results useful? [27].

## Results

**Fig. 1:**
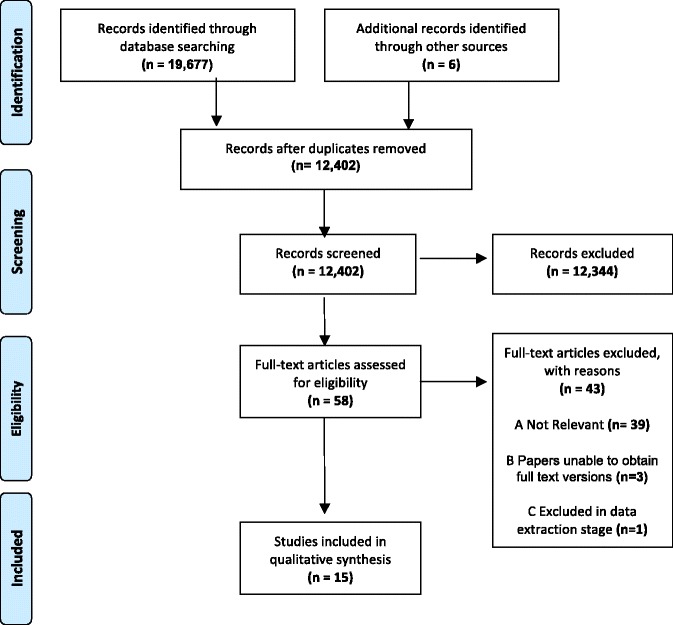
PRISMA flow chart showing the number of papers identified in the original literature searches and the study selection process

Figure 1 shows a PRISMA diagram displaying the number of papers excluded at each sifting stage [28, 29], whilst Table 3 presents a summary of each paper, their key findings and the results of quality appraisal.

**Table 3:** Summary table of included studies

Study	Intervention	Study design	Population	Implementation measurement	Data analysis	Key results—*factors affecting implementation*	Quality appraisal
Audrey et al., 2008 [34] UK	*Tobacco* A Stop Smoking In Schools Trial (ASSIST)	cRCT. questionnaire interviews	30 ASSIST schools & 29 control	Process evaluation to examine the context, implementation and receipt of the intervention	Framework method of data management. (reading, coding & identifying themes, & sorting material according to key issues)	Teachers welcomed external training—it interested pupils, prevented difficulties of discussing smoking with teachers and relieved staff burden. Implementation appeared compatible with the school ethos and timetable. Smoking was perceived as a difficult issue and staff welcomed a new initiative. Disruption to the timetable was inevitable, and the importance of communication between ASSIST staff and teachers was important	CASP: Moderate
Barr et al., 2002 [33] USA	*Tobacco* Tobacco Use Prevention Education (TUPE)	Telephone survey	296 middle school teachers & 282 high school teachers	Relations between TUPE teachers’ receptivity or amenability to implement TUPE programs and features of implementation settings	Cluster analyses for amenability to implementation. A one-way ANOVA for associations between amenability and implementation. A hierarchal multiple-regression for staff effectiveness perceptions	Indicators of staff amenability were variable. The most amenable staff reported consistently covering each activity with few barriers. For staff perceptions of effectiveness to prevent smoking initiation: Tobacco related norms accounted for 9.9% of variance, staff training & TUPE support or barriers—4.2%, and class activities—4.0%. For staff perceptions of TUPE for cessation: Tobacco norms—6.6% of variance, staff training & TUPE support—6.3%, class activities—5.5%	EPHPP: Moderate
Basen-Engquist et al., 1994 [31] USA	*Tobacco.* Minnesota Smoking Prevention (MSPP)	Questionnaire	39 districts in live training & 33 in video training. Mean number of pupils was 41, 2.8 teachers	Assessing how the type of teacher training affects implementation via a live workshop or video training	Fisher exact test & Mann-Whitney *U* for differences in teacher implementation Two group *t*-tests tested differences between students in the live and video districts	The relationship between type of training and use of the curriculum was significant. Districts who were assigned to the video training condition were less likely to teach the curriculum. However, implementing teachers from both groups reported high levels of implementation. Students in live workshops were more likely to recall discussions and activities	EPHPP: Weak
Garrahan 1995 [40] USA	*Substance Use*	Systems approach model	800 students	Not stated	Baseline substance use data was collected via a survey & analysed	Involving school personnel in a building-wide manner and monitoring efforts and outcomes was important. All implemented intervention aspects were linked to existing components of the school, and this gave the impression that what was implemented was based on common sense or self-evident reasoning	EPHPP: Weak
Jarrett et al., 2009 [35] USA	*Tobacco* Not-On-Tobacco (NOT)	Survey	769 pupils who reported regular smoking.	Perceptions of facilitator characteristics & the relationship between perceptions & outcomes	Descriptive analyses used to determine overall ranking of facilitator characteristics. Chi-square test to determine if facilitator ratings differed by race or sex	88.7% of pupils rated facilitators as favourable. No nagging or preaching, nonjudgmental, trustworthy, caring, & confidentiality were scored highly. There were few differences in ratings by race. Favorability scores were associated with changes in smoking (quit or reduce). Pupils who perceived facilitators favourably showed significant smoking reduction and cessation rates, regardless of sex or race	EPHPP: Weak
MacDonald and Green 2001 [44] Canada	*Substance Misuse*	Interviews and observations with Project Workers (PWs)	100 interviews in 6 sites with school admins, teachers, pupils, parents, & agency staff	Participants were probed around the level of understanding and support for prevention, implementation experiences, implementation barriers & facilitators, support for PWs and the school’s problem with drug and alcohol issues	Constant comparative method of grounded theory Field notes were recorded and used to support analysis	PWs needed to establish legitimacy and familiarity within schools, by overcoming staff opposition. They had to address conflicting expectations, resulting from poor preparation. Schools had to be ready and willing to implement, and PWs faced issues selling the model, and facilitating participation. Training sought to teach PWs to understand the model, but this did not occur and PWs realised they did not understand it enough to implement to others and few achieved it as intended. Some tried, but were discouraged by school barriers. Some retained key features, but omitted elements due to admin pressure or context demands	CASP: Strong
McBride et al., 2002 [43] Australia	*Alcohol* The School Health & Alcohol Harm Reduction Project (SHAHRP)	Longitudinal study	41 classes.28 teachers 6 schools	Series of methods to optimise and assess implementation fidelity including training, critical assessment and self report	Spearman’s rank measured fidelityTheme matrices described qualitative responses	SHAHRP was taught 80.7% as intended, with fidelity ranging from 78.9 to 83.4%. Implementation was optimised by: training, staff and pupil motivation and timing. Teachers found too much work in some lessons, interruptions reduced classroom time and implementation effectiveness was pupil dependent. Expectations needed to be lowered for difficult pupils and some activities were not implemented as intended	CASP: Weak EPHPP: Strong/Moderate
McCormick et al., 1995 [32] USA	*Tobacco*	RCT	21 districts, 50 schools, and 3000 pupils. Districts were assigned to control or intervention.	Use of ‘Level of use’ tool and implementation check-sheets	Population means, median, frequencies & correlations used for summary. Non-parametric tests tested for differences between control and intervention	Overall implementation completeness was low, with the mean % implemented being 70% and 23% implemented ≥90%. Larger districts were more likely to implement than small ones. Districts with favourable climates were more likely to implement and reported higher usage. Trained teachers were more likely to implement curricula and more likely to implement higher proportion	EPHPP: Moderate
Pettigrew et al., 2013 [42] USA	*Substance use* keepin’ it Real (kiR)	Ethnography	39 schools; 14 Control, 14 Rural: Mean number of pupils per school = 99, with a range from 27 to 226	An assessment of teacher implementation using the indicators; delivery methods, consistency of delivery, teaching standards	Coding provided; quantitative implementation ratings—quality adherence, adaptation, delivery and engagement, whilst qualitative codes identified adaptation and engagement	Analysis identified teacher control as passive, coordinated, or strict, and pupil participation as disconnected, attentive, or participatory; serving as a classroom typology for kiR implementation. Passive teachers were linked with passive pupils, strict teachers had attentive pupils, whilst classes with participatory pupils were taught by coordinated teachers. Teachers who taught kiR frequently tended to display similar control and pupils participated consistently	CASP: Moderate
Rohrbach et al., 2007 [37] USA	*Substance Use* Project Towards No Drug Abuse (TND)	RCT	18 schools—6 in each different condition. Pupils ranged from 13 to 19 years of age	Study compared teachers with Program Specialists (PSs). Questionnaire assessed implementation fidelity of TND via adherence, classroom process and perceived pupil acceptance	Inter-rater reliability was calculated for each item. To test the effect of implementer on fidelity and outcomes, a mixed-linear model was used	Of the 4 indexes of fidelity, only delivery quality differed between PSs and teachers. Both teachers and PSs achieved effects on 3 of the 5 immediate outcome measures, including program knowledge, addiction concern, and self-control. Pupils’ posttest ratings of the program and the quality of delivery showed no difference between teacher and specialist-led classrooms	EPHPP: Moderate
Skara et al., 2005 [36] USA	*Substance Use* Project Towards No Drug Abuse (TND).	Questionnaire	18 schools—6 in each different condition. 2735 students completed pretest questionnaires: 85% completed post-program	Questionnaire assessed implementation fidelity of TND via questions open and closed questions	Data was analysed using a generalised mixed-linear model using SAS	The curriculum was implemented as intended, received favourable ratings, and significantly improved knowledge. Providers reported high adherence to lesson plans and lessons were not difficult to teach. Adherence and delivery quality did not differ by curriculum or school. Individual ratings of delivery quality were favourable, including providers’ perceptions of pupil participation, pupil interest, provider’s maintenance of class control & providers’ perceptions of effectiveness	EPHPP: Moderate
Sloboda et al., 2009 [39] USA	*Substance Use* Take Charge of Your Life (TCYL)	Observation and surveys	TCYL was delivered by 140 Drug Abuse Resistance Education (DARE) officer instructors	Implementation fidelity measured using instructional strategy (IS)	Descriptive statistics & analyses between content coverage and IS & scores from targeted lessons were conducted using hierarchical linear modelling to gain 2-level random intercept models	Higher content was correlated with IS. There were no correlation between age, sex, race, education, content coverage or use of IS. Pupils with higher coverage scored higher on the consequences measure. Results indicated pupils with a higher proportion of the content had greater perceptions of negative consequences. Greater exposure and greater content coverage was related to negative alcohol expectancies	EPHPP: Weak
Stead et al., 2007 [38] UK	*Substance Use* Blueprint	Observations and interviews	30 schools in 4 Local Authority areas: 24 intervention & 6 control. Year 7 (11–12 years) & Year 8 (12–13 years)	Implementation fidelity measured via adherence, exposure, participant responsiveness, quality of delivery and program differentiation	Observation schedule used to generate descriptive statistics.	The mean content fidelity was 72%. As teachers got familiar with lessons, they were likely to modify or omit elements. Fidelity was highest in teacher-pupil lessons & lowest for pupil-pupil. Resource use was variable and teachers found timing and completing content difficult. Teachers were unsure of interactive sessions due to disruption & unpredictable outcomes. Some teachers expressed concern about answering questions about drugs, but there was no difference in delivery quality of teachers with experience & those without	CASP: Moderate
Sussman et al., 1993 [30] USA	*Tobacco* Project Towards No Tobacco Use	Questionnaire	4852 7th grade pupils. 9 Health Educators. 76 observers collected teacher data	Key implementation measures were around program completion and delivery (fidelity- adherence, exposure, reinvention)	Pupils & educators gave ratings of implementation. Post hoc comparisons were used between pairs of means and one-way ANOVAs predicted response means	Adherence did not vary by condition and high levels of implementation were observed in all conditions. Pupils preferred physical consequences and enthusiasm was rated the lowest. Health educators’ enthusiasm, effort and class enthusiasm differed by condition. Teachers did not differ in their ratings of class control or understandability	EPHPP: Weak
Thaker et al. 2008 [41] USA	*Substance Use* Reconnecting Youth (RY) program	Organisational diffusion study	At risk of drop out students from grades 9–12. 5 schools from each district took part	Three diffusion of innovation indicators used: perceived advantage, complexity and compatibility. Capacity, school turbulence and leadership/admin support were also explored to assess how they could affect implementation	Survey data was analysed using SPSS whereas interview data was transcribed and analysed using qualitative content analysis	Teachers reported learning RY difficult, as they were not prepared & needed to plan. RY was rigid, complex and difficult to implement the timelines & content. School capacity (skills and resources) varied & affected implementation. Other issues were budget shortfalls, funding cuts, difficulties finding rooms and school turbulence (transient pupil populations, school reorganisation, schedule changes, & staff turnover). RY lacked leadership and admin support. Only 50% of staff reported principles being supportive. Whilst only 1/3 of district admins considered RY important	EPHPP: Weak

Fifteen papers were deemed eligible for inclusion in the review. Six papers focused on tobacco interventions [30–35], four focused on drug use interventions [36–39], three focused on general substance use interventions [40–42], one focused on an alcohol intervention [43] and one focused on a dual alcohol and drug intervention [44]. All but one paper lacked the use of an implementation theory [44, 45] and no reference was made within the included papers to the use of implementation strategies.

### Results of quality assessment

The results of the quality assessment are displayed in the final column of Table 3. The EPHPP tool identified that, of the quantitative papers, four were moderate quality papers [32, 33, 36, 37] and seven were classed as weak papers [30, 31, 35, 39–41, 43]. The weakest areas in papers included validity and reliability of data collection, reporting of participant withdrawals, and nearly every paper lacked confounding factor reporting. The five qualitative papers were rated, using CASP, from strongest to weakest by how many ‘Yes’ , ‘No’ or ‘Can’t tell’ outcomes they received. The weakest papers lacked reporting of specific methodological details and no papers made reference to ethical considerations or obtaining ethical approval.

### Synthesis of results

During data extraction, the key factors, found to affect the implementation of a tobacco or substance use intervention within a secondary school, were identified within the 15 included papers and coded and organised using the four NPT constructs**.** This is displayed in Table 4.

**Table 4:** A summary of the key results organised by their corresponding NPT construct

Factors affecting implementation	Papers	NPT construct
Distinguishing from current practice	[34, 40]	Coherence
Fitting with school ethos	[34]	Coherence
Providers seeing the value or benefit of an intervention	[34, 36, 38, 44]	Coherence
Providers not delivering or not understanding how to deliver (use of specialist knowledge)	[38, 41, 42, 44]	Coherence Collective Action
Training	[32, 34, 41, 42]	Coherence Collective Action
Implementation driving force	[34, 37, 42–44]	Cognitive Participation
Role identity—provider ‘agreeing’ it should be part of their role	[30, 34, 40, 43, 44]	Cognitive Participation
Provider supporting intervention	[30, 33, 34, 39, 41]	Cognitive Participation
Provider motivation	[43]	Cognitive Participation
Sustainability	[30]	Cognitive Participation
Young people behaviour	[42]	Cognitive Participation
Providers feeling uncomfortable with delivery	[38]	Cognitive Participation Collective Action
Budget cuts or limited resources	[41]	Collective Action
Disruption to school timetable	[34]	Collective Action
Favourable organisational climate/host support	[32, 34, 40, 41, 44]	Collective Action
Fidelity	[30, 31, 33, 36–39, 41–44]	Collective Action
Importance of staff skills, knowledge or characteristics	[35, 42]	Collective Action
Involving schools; monitoring outcomes	[40]	Collective Action
Schools prepared for implementation	[44]	Collective Action
Staff turnover	[41]	Collective Action
Modifying practice (from feedback)	[38]	Reflexive monitoring
Negative implementation experience	[41]	Reflexive monitoring
Positive feedback	[36]	Reflexive monitoring

### Coherence

The coherence construct of NPT refers to the sense-making work that individuals participate in either individually, or collectively, when operationalizing a new intervention [25]. A key result relating to Coherence was that providers were often found to not understand, or were not able to make sense of what a tobacco or substance use intervention required, in order to implement it successfully [38, 41, 42, 44]. MacDonald and Green found that Project Workers (PWs) responsible for implementing their substance use intervention *‘* *didn’t* *understand* *the* *model* *enough* *to* *implement* *it* *or* *to* *sell* *it* *to* *others*’ [44]*.* PWs were unable to make sense of the intervention and therefore were unable to fulfil their role of introducing and implementing the intervention [44]. This was reported similarly in the paper by Thaker et al., as learning the Reconnecting Youth (RY) intervention was found to be challenging, and even following training, teachers found RY to be complex and difficult to implement [41].

Training was identified in a large proportion of included papers as a factor with the potential to facilitate implementation within the secondary school setting [32, 34, 41, 42]. Specific examples included McCormick et al. identifying that teachers who were adequately trained to deliver their tobacco intervention were more likely to implement curricula, and also increased the amount of curricula implemented [32], whilst Pettigrew et al. reported that the training, that was provided for the implementation of their substance use intervention, was insufficient for maintaining implementation fidelity and improving outcomes, and the importance of investment in delivery personnel, and delivery support was emphasised [42]. Basen-Engquist investigated the effect on implementation of a tobacco intervention when providers were trained in a live session in comparison to video training [31]. They reported that providers in the video training condition were less likely to teach the curriculum, indicating pre-recorded training affected implementation [31]. Sloboda et al. showed higher content coverage was correlated with appropriate instructional strategy (*r* = 0.93, *P* < 0.001). In Stead et al., some teachers were new and were concerned with implementing the substance use intervention as required [38]. Although training emphasised that teachers did not need specialist drug education, some felt uncomfortable about being unable to answer students’ questions [38].

The ability of participants to distinguish the intervention from their current ways of working was also identified as being a factor affecting implementation [34, 40]*.* Audrey et al. reported that as smoking was seen as problematic in schools, secondary school staff welcomed the implementation of a tobacco intervention, that was different from their current practices [34]. But due to the heterogeneity of the results, it was also identified to remain cautious when straying considerably from existing practice, as Garrahan reported that all of their intervention elements were linked to existing school components as ‘*it* *gave* *the* *impression* *that* *much* *of* *what* *was* *done* *was* *based* *on* *common* *sense* *or* *derived* *by* *reasoning* *from* *self-evident* *conditions*’ [40].

It was identified as being important for tobacco or substance use interventions to fit with a school’s ethos, in order to be able to construct a degree of value to implement [34, 36, 38, 44]. A specific example of this was Audrey et al. reporting the importance of using peer students, as it resulted in the recruitment of students representative of their peer group and staff found this to be valuable [34].

### Cognitive Participation

In the context of this review, the construct Cognitive Participation was used to refer to the relational work that individuals do to build and then sustain a community of practice around a new intervention within the secondary school setting [25].

Having a designated individual or a group of individuals to act as implementation driving forces was identified in several papers as being important in influencing the implementation of a tobacco or substance use intervention [34, 37, 42–44]. A specific example included Audrey et al., which reported the importance of students in driving implementation and in engaging other peers to be involved with the intervention [34]. This is also linked with provider motivation and buy-in. McBride et al. discusses teachers’ motivation and their view of students’ motivation towards the alcohol intervention SHAHRP. Motivation positively influenced teachers’ willingness and commitment to implement as intended, as there was buy-in and support for the intervention in response to students’ attitudes [43]. Motivated teachers were seen to act as implementation driving forces in which to motivate students [43]. This was further confirmed by Rohrbach et al., as ‘*motivated,* *trained* *classroom* *teachers*’ implemented substance use programs with fidelity and achieved immediate effects [37]. In Sussman et al., it was reported that health educators’ enthusiasm, effort and class enthusiasm differed, when it came to the implementation of their tobacco intervention, indicating there was differing levels of willingness depending on the context [30].

Pettigrew et al. acknowledged that whilst teachers played a central role in driving the intervention implementation, students’ behaviour was important, as not all students appeared equally engaged. Some displayed disconnected behaviour, whilst others were attentive or participatory, and this affected implementation [42]. In addition, in the paper by Jarrett et al., an association between teens’ perceptions of facilitator characteristics and how important N-O-T was in quitting smoking was reported [35].

The perceptions of providers, and agreeing that a tobacco or substance use intervention should be part of their work were identified as factors affecting implementation [30, 34, 40, 43, 44]. This was seen in Audrey et al., as teachers recognised the importance of using student peers, as it ‘*complemented* *their* *attempts* *to* *promote* *confidence* *and* *responsibility*’ [34]. In Barr et al., results showed that teachers’ perceptions of the implementation settings significantly influenced their reactions, which ultimately affected implementation and long-term sustainability [33]. Sustainability was also discussed in Macdonald and Green as PWs needed to maintain willingness to introduce and implement new practices, and sustainability was often difficult [44]. The paper by Stead et al. reported tension, with teachers being uncomfortable with some of the intervention sessions [38]. Sessions, such as interactive sessions, were not looked upon favourably by some providers and were therefore not delivered as intended, indicating providers were less likely to agree that an intervention should be part of their work if they were unhappy or uncomfortable with delivery [38].

### Collective Action

The construct Collective Action characterises the operational work that individuals are required to do in order to be able to enact a new practice [25]. Fidelity, or how closely an intervention is implemented as intended, was one of the most commonly discussed factors affecting implementation within included papers [30, 31, 33, 36–39, 41–44]. Fidelity generally appeared high across the papers, with McBride et al. reporting 80.7% of SHAHRP was taught as intended, in Rohrbach et al., out of four implementation indexes, only one showed differences in delivery between program specialists and teachers, and both Sloboda et al. and Skara et al. reported programs being implemented as intended [39]. In Thaker et al., RY was implemented according to protocols, and high fidelity was observed in all schools [41]. Sussman et al. reported adherence did not vary by condition, and high fidelity was observed in all conditions [30]. Basen-Engquist et al. reported teachers from both groups reported high implementation fidelity [31]. Pettigrew et al. found that teachers, who taught the kiR intervention more than once, tended to exert similar levels of control in delivering curriculum and students exhibited consistent participation levels [42].

However, high fidelity was not observed in Barr et al. as it reported substantial heterogeneity in teachers’ amenability and tasks [33]. Stead et al. reported the mean lesson content fidelity to be 72%, but as teachers became familiar with lessons they were more likely to modify or omit elements [38]. MacDonald and Green reported that few PWs were able to implement the model as intended [44]. Some PWs reported trying to, but were discouraged by school barriers and administrative pressures, indicating inadequate support from the school acted as a factor negatively affecting implementation [44].

This links to the several papers identifying ways in which contextual factors affected implementation [32, 34, 40, 41, 44]. MacDonald and Green reported that before PWs could implement new strategies, schools needed to be ready and willing [44]. Issues were reported with selling the program, facilitating participation, and also steering the committee, indicating host support was lacking [44]. Further challenges were reported in Thaker et al., with only 50% of staff reporting they had Head Teacher support [41]. Teachers in one school reported that the assistant principal and counsellors did not support RY and support for student recruitment and teachers was also lacking [41]*.* In addition, the capacity of skilled staff and resources varied significantly. Budget shortfalls, funding cuts and inadequate resources, such as classroom space, were all cited as factors negatively affecting implementation [41]. Garrahan emphasised the importance of involving school personnel in a building-wide manner, and monitoring efforts to achieve outcomes were found to be beneficial [40].

Timing was reported as a factor negatively affecting implementation. McBride et al. reported that teachers found it difficult to complete activities in the allocated time, as did Thaker et al. [41, 43]. This was also observed in Stead et al., where teachers frequently overran and lacked sufficient preparation time, indicating the operational work was not appropriately allocated [38]. Audrey et al. also presented findings around the allocation of work during implementation, with teachers welcoming training by external trainers, as it created a greater interest amongst students and reduced the difficulties facing students discussing smoking with teachers [34].

Thaker et al. reported a high level of staff turnover as a factor negatively affecting implementation, as teachers reported that staff turnover made the implementation of substance use interventions difficult, and it made it difficult for providers to maintain trust in each other’s work [41]. Trust and communication were also identified as being factors facilitating implementation in Audrey et al. The implementation of ASSIST was responsible for causing disruption to the school timetable, with students needing to be removed from classes. This was ameliorated by facilitating communication between the ASSIST team and school contacts, and between teachers within the school [34].

### Reflexive Monitoring

Reflexive Monitoring considers the appraisal work that individuals participate in to assess and understand the ways that a new practice can affect them and the others around them [25]. Few of the included papers reported results indicative of the Reflexive Monitoring construct; only one paper reported participants modifying work in response to intervention appraisal [38], and there was a general lack of evaluatory components or reporting of how participants appraised implementation and how to improve the process.

In Skara et al., providers gave delivery quality ratings, such as their perception of student participation. As this was high (*M* = 6.2 on 7 point scale), delivery quality was reported as ‘very favourable’, indicating participants evaluated the implementation of the substance use intervention positively [36]. In Stead et al., the amount of activities in the implementation of Blueprint were modified, as a result of teacher feedback. Feedback highlighted that there was insufficient time to cover all aspects as intended, and although developers reduced the content, lessons still remained content rich and timing remained problematic [38]. One school, in Thaker et al’s study, evaluated the implementation of RY extremely negatively and stated they would be unlikely to implement RY again due to ‘a lack of flexibility, high preparation and a bad implementation experience’ [41].

## Discussion

Despite the 15 included papers being heterogeneous, common factors affecting the implementation of tobacco and substance use interventions in the secondary school could be identified. During quality appraisal, the majority of papers were classified as weak or moderate quality. A common weak area was found to be the reporting of confounding and contextual factors affecting study results, which was also identified in the review of healthcare innovation by Greenhalgh et al. [18]. By offering more of a focus to confounding factors, which have the potential to affect implementation, it is likely to add value in providing a richer understanding of the context and facilitate implementation within the secondary school setting.

This is a common thread within the implementation field, and advances in implementation science has led to the identification that implementation studies often display insufficient and inadequate reporting, requiring intervention [46–48]. Therefore, the recently published Standards for Reporting Implementation Studies (StaRI) Statement was developed as a set of guidelines to increase the transparency and accuracy of implementation study reporting [46]. This would particularly be of use within the school setting, as the reporting was shown in the review to be largely disparate. By employing the StaRI guidelines, within future implementation studies in the school setting, it would likely have a significant impact on the structure and reporting of implementation outcomes, and would not only ensure the delivery of higher quality papers but would increase the comparability and work towards improving implementation in practice in this setting [46].

NPT was used to provide a common interpretative framework to apply across the full set of papers and ensured that a comprehensive assessment of the factors affecting implementation could be made. This sought to be a novel element of this paper as NPT’s use outside of the healthcare setting has been limited, and no previously published work has used it within the secondary school context. This has implications for broader implementation research, as it emphasises the usefulness of NPT in the school setting, and highlights the transferability of NPT in settings outside of healthcare.

A key result, relating to the implementation determinants of tobacco and substance use interventions, was that few papers reported providers being able to distinguish the intervention from their current ways of working*.* This is likely to create difficulties with staff engagement, which was also reported as a key factor affecting implementation, as there is no clear discernible benefit to a new practice. However, if an intervention is highly removed from current practice, providers may struggle with role identity conflicts, if it is perceived as being outside their traditional role. This is of increased importance within the secondary school setting, as staff in the included papers reported heavy workloads and time pressures, indicating the adoption of a new role or practice may provide a degree of conflict.

Another key factor determining implementation was the providers’ level of comfort with delivery and the topic. This has not been observed in a similar way in general health promotion implementation studies in schools [15] but is largely unsurprising when focusing on tobacco or substance use interventions, as they are often associated with negative stigma [49, 50] indicating a consideration for future work. Results highlighted providers feeling unprepared or that specialist knowledge was required to deliver interventions effectively [44]. This links with the conflict around role identity and the importance of training, which was emphasised in several papers [32, 34, 41, 42]. Comprehensive training can therefore be highlighted as an implementation strategy, which can positively affect implementation, if it is able to address how to deliver controversial topics and leaves providers feeling adequately supported.

Support and provider buy-in were consistently portrayed as factors facilitating and determining implementation and good engagement were seen to positively influence student behaviour [30]. In addition, provider support was linked with the need for an implementation driving force. Due to the disparate nature of the papers and their context, this was explored differently, with students, teachers, project workers and outsider providers acting as implementation drivers. Organisational support, which has previously been identified as a key implementation determinant [45, 51, 52], was also identified as a key factor affecting implementation, with the most effective support being gained pre-implementation and providing long-term maintenance [32].

Another result specific to the school setting included student engagement, which was observed as a factor affecting implementation in Pettigrew et al. [42]. Although the school settings were shown to be highly heterogeneous, this is likely to be common across schools, as individual differences will affect students’ engagement levels.

Moving on to consider the finding around the implementation outcome fidelity, implementation fidelity appeared to be variable across the included papers and was affected by multiple factors. In some papers, providers felt it necessary to modify intervention components, leading to emphasising the importance of establishing which components are essential for implementation and which components should possess flexibility. Implementation fidelity is often considered as being complex and a key source of variability [37, 53, 54]. A specific example from the surrounding literature is within the review of implementation fidelity of school-based drug use interventions by Dusenbury et al. [55]. The idea that school providers can reduce implementation fidelity, but ultimately increase the ‘implementability’ , is an important area to discuss. Although, it was seen as beneficial to possess flexibility, as programs that were too rigid experienced low fidelity, it is important to identify critical intervention components, to ensure that modifications do not affect the intervention’s effectiveness. Therefore, to facilitate future school-based tobacco or substance use intervention implementation, core elements should be identified and complemented with flexible components, in order to be salient for the differing school contexts. This observation is also supported by several papers reporting teachers struggling with adhering to timelines; as although teachers were seen to be appropriate providers, heavy workloads made it difficult to maintain fidelity due to the preparation or time constraints. It is likely to be inappropriate to allocate teachers large implementation activities, and it may prove advantageous, if feasible, to source training or delivery to outside providers.

Even though this review highlights factors unique to the school setting, such as provider factors and pupil engagement, fitting this review into the wider implementation literature context; the findings around organisational host support, adequate resources and the need for appropriate feedback echo the findings of previously conducted implementation work [14, 45, 56, 57]. NPT was useful as an organising framework for synthesising findings from disparate study designs, to not only identify the factors affecting implementation, but also to highlight the knowledge gaps and areas warranting future research and or action in terms of intervention modification.

A unique finding of the review was that few of the included papers reported results indicative of NPT’s Reflexive Monitoring construct. This could have resulted from methodological reasons, such as participants were not asked or the intervention effects were not known, or could simply be a result of the previously discussed limited reporting. However, as evaluations can provide value to implementation studies by identifying ineffective areas, such as provider or host support, it is likely that building in feedback or evaluation components into future work in the school setting would be advantageous.

Other gaps included papers lacking reporting around the use of predefined implementation strategies, which can be complemented by the use of implementation theory. As stated, almost all of the review’s included papers lacked a theoretical driving mechanism. We argue that future school implementation work would significantly benefit from being theoretically driven, and this has frequently been raised when considering existing implementation studies [46, 58–60]. By employing the use of a conceptual framework to underpin the implementation research in the secondary school setting, it could have facilitated implementation strategies and the reproducibility and clearly highlighted specific areas of improvement for future implementation and sustainability.

This finding has broad implications for future work, and one of the goals of this systematic review has been to inform the development of a school-based intervention implementation model to facilitate the implementation of novel substance use interventions in the secondary school setting. Although the model will be developed with reference to the rapidly advancing knowledge on implementation determinants assessment [61] implementation strategies [62] and progress and outcomes assessment and measurement [63], it will be informed by in-depth qualitative research currently being undertaken with local school staff and key stakeholders in the implementation process to ensure targeting of key challenges in the secondary school setting. This review thus represents initial advancement in understanding the challenges of implementing substance use interventions in the school setting, as part of a programme of work that moves more towards the development and testing of tools for facilitating improved implementation of such interventions. Conceptual and practical developments stemming from this work will therefore be useful in the wider school implementation field and will be publicly available for use in future implementation research in this setting.

A final gap identified was there was little to no focus, within the included studies, around the cost effectiveness of implementation. This could benefit from playing a role in future work as small budgets and cuts to school funding were reported to be factors negatively affecting the implementation of a tobacco or substance use intervention, specifically within the context of UK secondary schools [41]. There remains limited available research evidence investigating how altering the implementation of a such an intervention, could influence the total cost, and which costs can be directly attributable to implementation. Therefore, as the secondary school setting remains to be a financially restricted setting, it highlights a key area of investigation for school-based intervention implementation research and one which will be explored within the future planned work.

### Limitations

Although systematic search procedures were followed, it is possible that key studies were missed, or published after searching concluded. However, the authors minimised the likelihood of this by double sifting, reference list searching and re-running searches during the period of research.

The included papers were highly heterogeneous, making synthesis and interpretation of authors’ findings challenging. NPT did, however, provide a common framework against which to link and synthesise study findings, and best practice approaches to narrative synthesis (including multiple team member checking of data interpretation) add to our confidence in the presentation of findings. Our findings in relation to policy and practice at this moment in time should thus be deemed as tentative, but will be further explored in in-depth qualitative research with key stakeholders.

We acknowledge that other implementation theories or frameworks could have been employed differently to further classify and interpret the results. NPT was most useful for the purpose of our review, given the small but diverse literature we synthesised. However, a more elaborate tool such as that offered by Flottorp et al. could be used to map existing theories by their corresponding constructs and is likely to be useful future reviews in this field [61].

## Conclusion

This review identified and synthesised factors reported to affect the implementation of tobacco and substance use interventions within the secondary school setting. Key factors affecting implementation that were identified, such as contextual factors, and support and training and provider perceptions, should be understood and addressed when implementing secondary school-based interventions. However, increased exploration should be provided to NPT’s reflexive monitoring construct, the appraisal and evaluation processes of implementing new interventions, as findings around providers reflecting upon components they believe facilitated the implementation process and which aspects could benefit from modifications, were limited and are likely to add value in facilitating improved implementation and sustainability of interventions in the future.

This review sought to reinforce the importance of considering the factors affecting introducing a new intervention into practice. As there were relatively few papers specifically focusing on the implementation of tobacco or substance use interventions in the secondary school, it demonstrated that the school health field could benefit from more work in this area and should build on the findings and lessons from the existing school implementation work. Research should focus on bridging the gap between research and practice, and reflective collaborative working involving educators and practitioners will be conducted, in order to generate an implementation model with the most salience for this setting. Working collaboratively to develop implementation strategies, which employ the use of implementation theory and which comprehensively consider the implementation outcomes, such as adoption, feasibility and acceptability in practice, would be advantageous and would likely contribute to increasing the effectiveness of interventions seeking to reduce tobacco and substance use in adolescents.

## Additional file

Additional file 1: Data extraction worksheet. (XLSX 52 kb)

## Abbreviations

ANOVA: Analysis of variable
ASSIST: A Stop Smoking In Schools Trial
CASP: Critical Appraisal Skills Programme
CINAHL: Cumulative Index to Nursing and Allied Health Literature
cRCT: Cluster Randomised Controlled Trial
EPHPP: Effective Public Health Practice Project
ERIC: Education Resource Information Centre
IS: Instructional Strategy
kiR: Keeping It Real
MSPP: Minnesota Smoking Prevention
N-O-T: Not on Tobacco
NPT: Normalisation Process Theory
PS: Program Specialist
RCT: Randomised Controlled Trial
RY: Reconnecting Youth
SHAHRP: School Health and Alcohol Harm Reduction Project
TND: Towards No Drug Use
TUPE: Tobacco Use Prevention Education
WHO: World Health Organization

## References

[1] Viner RM, Ozer EM, Denny S, Marmot M, Resnick M, Fatusi A (2012). Adolescence and the social determinants of health. Lancet.

[2] Santelli JS, Baldwin W, Heitel J (2015). Rising wealth, improving health? Adolescents and inequality. Lancet.

[3] Umberson D, Crosnoe R, Reczek C (2010). Social relationships and health behavior across life course. Annu Rev Rociology.

[4] Lake AA, Adamson AJ, Craigie AM, Rugg-Gunn AJ, Mathers JC (2009). Tracking of dietary intake and factors associated with dietary change from early adolescence to adulthood: the ASH30 study. Obesity Facts.

[5] Kratochwill TR, Albers CA, Shernoff ES (2004). School-based interventions. Child Adolesc Psychiatr Clin N Am.

[6] Botvin GJ, Griffin KW (2007). School-based programmes to prevent alcohol, tobacco and other drug use. Int Rev Psychiatry.

[7] Newbury-Birch D, Scott S, O’Donnell A, Coulton S, Howel D, McColl E, Stamp E, Graybill E, Gilvarry E, Laing K, McGovern R. A pilot feasibility cluster randomised controlled trial of screening and brief alcohol intervention to prevent hazardous drinking in young people aged 14–15 years in a high school setting (SIPS JR-HIGH). Public Health Research. 2014;2(6). 25642576

[8] Hurrelmann K, Richter M (2006). Risk behaviour in adolescence: the relationship between developmental and health problems. J Public Health.

[9] WHO. The World Health Organisation, Adolescent Development 2015 Available from: http://www.who.int/maternal_child_adolescent/topics/adolescence/dev/en/. Accessed 9 July 2016.

[10] Steinberg L (2007). Risk taking in adolescence new perspectives from brain and behavioral science. Curr Dir Psychol Sci.

[11] Selemon LD (2013). A role for synaptic plasticity in the adolescent development of executive function. Transl Psychiatry.

[12] Foxcroft DR, Tsertsvadze A (2011). Cochrane review: universal school-based prevention programs for alcohol misuse in young people. Evid Based Child Health.

[13] Glasgow RE, Lichtenstein E, Marcus AC (2003). Why don’t we see more translation of health promotion research to practice? Rethinking the efficacy-to-effectiveness transition. Am J Public Health.

[14] Domitrovich CE, Bradshaw CP, Poduska JM, Hoagwood K, Buckley JA, Olin S (2008). Maximizing the implementation quality of evidence-based preventive interventions in schools: a conceptual framework. Adv School Ment Health Promot.

[15] Pearson M, Chilton R, Wyatt K, Abraham C, Ford T, Woods H (2015). Implementing health promotion programmes in schools: a realist systematic review of research and experience in the United Kingdom. Implement Sci.

[16] May C, Finch T (2009). Implementing, embedding, and integrating practices: an outline of normalization process theory. Sociology.

[17] Murray E, Treweek S, Pope C, MacFarlane A, Ballini L, Dowrick C (2010). Normalisation process theory: a framework for developing, evaluating and implementing complex interventions. BMC Med.

[18] Greenhalgh T, Robert G, Macfarlane F, Bate P, Kyriakidou O (2004). Diffusion of innovations in service organizations: systematic review and recommendations. Milbank Q.

[19] Damschroder LJ, Aron DC, Keith RE, Kirsh SR, Alexander JA, Lowery JC (2009). Fostering implementation of health services research findings into practice: a consolidated framework for advancing implementation science. Implement Sci.

[20] Greenberg MT, Domitrovich CE, Graczyk PA, Zins J (2005). The study of implementation in school-based preventive interventions: theory, research, and practice. Promotion of mental health and prevention of mental and behavioral disorders 2005 series V3.

[21] May CR, Mair F, Finch T, MacFarlane A, Dowrick C, Treweek S (2009). Development of a theory of implementation and integration: normalization process theory. Implement Sci.

[22] May CR, Finch T, Ballini L, MacFarlane A, Mair F, Murray E (2011). Evaluating complex interventions and health technologies using normalization process theory: development of a simplified approach and web-enabled toolkit. BMC Health Serv Res.

[23] Mair FS, May C, O’Donnell C, Finch T, Sullivan F, Murray E (2012). Factors that promote or inhibit the implementation of E-health systems: an explanatory systematic review. Bull World Health Organ.

[24] O’Reilly P, Lee SH, O’Sullivan M, Cullen W, Kennedy C, MacFarlane A (2017). Assessing the facilitators and barriers of interdisciplinary team working in primary care using normalisation process theory: an integrative review. PLoS One.

[25] May C, Rapley T, Mair FS, Treweek S, Murray E, Ballini L (2015). Normalization process theory on-line users’ manual, toolkit and NoMAD instrument.

[26] EPHPP. Effective Public Health Practice Project- Quality Assessment for Quantitative Studies 2016 [Available from: http://www.ephpp.ca/tools.html]. Accessed 27 June 2016.

[27] CASP. Critical Appraisal Skills Programme- Quality Assessment Checklists Oxford2014 [Available from: http://www.casp-uk.net/casp-tools-checklists]. Accessed 27 June 2016.

[28] Liberati A, Altman DG, Tetzlaff J, Mulrow C, Gøtzsche PC, Ioannidis JP (2009). The PRISMA statement for reporting systematic reviews and meta-analyses of studies that evaluate health care interventions: explanation and elaboration. Ann Intern Med.

[29] Moher D, Liberati A, Tetzlaff J, Altman DG, Group P (2010). Preferred reporting items for systematic reviews and meta-analyses: the PRISMA statement. Int J Surg.

[30] Sussman S, Dent CW, Stacy AW, Hodgson CS, Burton D, Flay BR (1993). Project towards no tobacco use: implementation, process and post-test knowledge evaluation. Health Educ Res.

[31] Basen-Engquist K, O’Hara-Tompkins N, Lovato CY, Lewis MJ, Parcel GS, Gingiss P (1994). The effect of two types of teacher training on implementation of smart choices: a tobacco prevention curriculum. J Sch Health.

[32] McCormick LK, Steckler AB, McLeroy KR (1995). Diffusion of innovations in schools: a study of adoption and implementation of school-based tobacco prevention curriculum. Am J Health Promot.

[33] Barr JE, Tubman JG, Montgomery MJ, Soza-Vento RM (2002). Amenability and implementation in secondary school antitobacco programs. Am J Health Behav.

[34] Audrey S, Holliday J, Campbell R (2008). Commitment and compatibility: Teachers’ perspectives on the implementation of an effective school-based, peer-led smoking intervention. Health Educ J.

[35] Jarrett T, Horn K, Zhang J (2009). Teen perceptions of facilitator characteristics in a school-based smoking cessation program. J Sch Health.

[36] Skara S, Rohrbach LA, Sun P, Sussman S (2005). An evaluation of the fidelity of implementation of a school-based drug abuse prevention program: project toward no drug abuse (TND). J Drug Educ.

[37] Rohrbach LA, Dent CW, Skara S, Sun P, Sussman S (2007). Fidelity of implementation in project towards no drug abuse (TND): a comparison of classroom teachers and program specialists. Prev Sci.

[38] Stead M, Stradling R, MacNeil M, MacKintosh AM, Minty S (2007). Implementation evaluation of the blueprint multi-component drug prevention programme: fidelity of school component delivery. Drug Alcohol Rev..

[39] Sloboda Z, Stephens P, Pyakuryal A, Teasdale B, Stephens RC, Hawthorne RD (2009). Implementation fidelity: the experience of the adolescent substance abuse prevention study. Health Educ Res.

[40] Garrahan DP (1995). The application of a systems approach to substance use prevention: linking interventions to the infrastructure. J Alcohol Drug Educ.

[41] Thaker S, Steckler A, Sanchez V, Khatapoush S, Rose J, Hallfors DD (2008). Program characteristics and organizational factors affecting the implementation of a school-based indicated prevention program. Health Educ Res.

[42] Pettigrew J, Miller-Day M, Shin YJ, Hecht ML, Krieger JL, Graham JW (2013). Describing teacher-student interactions: a qualitative assessment of teacher implementation of the 7th grade keepin’ it REAL substance use intervention. Am J Community Psychol.

[43] McBride N, Farringdon F, Midford R (2002). Implementing a school drug education programme: reflections on fidelity. Int J Health Promot Educ.

[44] MacDonald MA, Green LW (2001). Reconciling concept and context: the dilemma of implementation in school-based health promotion. Health Educ Behav.

[45] Durlak JA, DuPre EP (2008). Implementation matters: a review of research on the influence of implementation on program outcomes and the factors affecting implementation. Am J Community Psychol.

[46] Pinnock H, Barwick M, Carpenter CR, Eldridge S, Grandes G, Griffiths CJ (2017). Standards for reporting implementation studies (StaRI) statement. Br Med J.

[47] Davies P, Walker AE, Grimshaw JM (2010). A systematic review of the use of theory in the design of guideline dissemination and implementation strategies and interpretation of the results of rigorous evaluations. Implement Sci.

[48] Proctor EK, Powell BJ, McMillen JC (2013). Implementation strategies: recommendations for specifying and reporting. Implement Sci.

[49] Stormshak EA, Dishion TJ, Light J, Yasui M (2005). Implementing family-centered interventions within the public middle school: linking service delivery to change in student problem behavior. J Abnorm Child Psychol.

[50] Luoma JB, Twohig MP, Waltz T, Hayes SC, Roget N, Padilla M (2007). An investigation of stigma in individuals receiving treatment for substance abuse. Addict Behav.

[51] Chaudoir SR, Dugan AG, Barr CH (2013). Measuring factors affecting implementation of health innovations: a systematic review of structural, organizational, provider, patient, and innovation level measures. Implement Sci.

[52] Weiner BJ (2009). A theory of organizational readiness for change. Implement Sci.

[53] Carroll C, Patterson M, Wood S, Booth A, Rick J, Balain S (2007). A conceptual framework for implementation fidelity. Implement Sci.

[54] Gingiss PM, Roberts-Gray C, Boerm M (2006). Bridge-it: a system for predicting implementation fidelity for school-based tobacco prevention programs. Prev Sci.

[55] Dusenbury L, Brannigan R, Falco M, Hansen WB (2003). A review of research on fidelity of implementation: implications for drug abuse prevention in school settings. Health Educ Res.

[56] Walker HM (2004). Commentary: use of evidence-based interventions in schools: where we’ve been, where we are, and where we need to go. Sch Psychol Rev.

[57] Kilbourne AM, Neumann MS, Pincus HA, Bauer MS, Stall R (2007). Implementing evidence-based interventions in health care: application of the replicating effective programs framework. Implement Sci.

[58] Eccles MP, Armstrong D, Baker R, Cleary K, Davies H, Davies S (2009). An implementation research agenda. Implement Sci.

[59] French SD, Green SE, O’Connor DA, McKenzie JE, Francis JJ, Michie S (2012). Developing theory-informed behaviour change interventions to implement evidence into practice: a systematic approach using the theoretical domains framework. Implement Sci.

[60] McEvoy R, Ballini L, Maltoni S, O’Donnell CA, Mair FS, MacFarlane A (2014). A qualitative systematic review of studies using the normalization process theory to research implementation processes. Implement Sci.

[61] Flottorp SA, Oxman AD, Krause J, Musila NR, Wensing M, Godycki-Cwirko M (2013). A checklist for identifying determinants of practice: a systematic review and synthesis of frameworks and taxonomies of factors that prevent or enable improvements in healthcare professional practice. Implement Sci.

[62] Powell BJ, Waltz TJ, Chinman MJ, Damschroder LJ, Smith JL, Matthieu MM (2015). A refined compilation of implementation strategies: results from the Expert Recommendations for Implementing Change (ERIC) project. Implement Sci.

[63] Lewis CC, Fischer S, Weiner BJ, Stanick C, Kim M, Martinez RG (2015). Outcomes for implementation science: an enhanced systematic review of instruments using evidence-based rating criteria. Implement Sci.

